# Factors Associated With the Use of the Lifestyle and Empowerment Techniques in Survivorship of Gynecologic Oncology (LETSGO) mHealth App in Routine Follow-Up After Gynecologic Cancer Treatment: Observational Study

**DOI:** 10.2196/89918

**Published:** 2026-07-31

**Authors:** Zaklina Tarabar, Elin Børøsund, Sindre H Fosstveit, Sveinung Berntsen, Milada Hagen, Ingvild Vistad

**Affiliations:** 1 Hospital of Southern Norway, Egsveien 100, Kristiansand, Agder, 4615, Norway, 47 91840889; 2 Institute of Clinical Medicine, Faculty of Medicine, University of Oslo, Oslo, Norway; 3 Department of Digital Health Research, Division of Medicine, Oslo University Hospital, Oslo, Norway; 4 Department of Nursing and Health Sciences, Faculty of Health and Social Sciences, University of South-Eastern Norway, Drammen, Norway; 5 Department of Sport Science and Physical Education, University of Agder, Kristiansand, Agder, Norway; 6 Research Unit, Sorlandet Hospital, Kristiansand, Norway; 7 Department of Nursing and Health Promotion, Oslo Metropolitan University, Oslo, Norway; 8 Department of Obstetrics and Gynecology, Sorlandet Hospital, Kristiansand, Agder, Norway

**Keywords:** app use, cancer survivors, digital follow-up care, digital health tools, mobile health apps, user engagement.

## Abstract

**Background:**

Mobile health (mHealth) apps offer new opportunities to support cancer survivors in managing their health, adopting healthier lifestyles, and increasing awareness of recurrence symptoms. However, despite their potential, little is known about survivors’ use of such tools in routine follow-up care, or which factors are associated with their engagement.

**Objective:**

The aim of the present study was to identify survivor characteristics associated with the use of the Lifestyle and Empowerment Techniques in Survivorship of Gynecologic Oncology (LETSGO) app. In addition, we aimed to describe engagement patterns among gynecologic cancer survivors during the first year of follow-up after treatment.

**Methods:**

App data from 378 participants in the intervention group of the LETSGO multicenter clinical trial were included in the analysis. Using multiple logistic regression, we analyzed app data from the first year of LETSGO app use to identify participant characteristics associated with use. In addition, we described the frequency of user interactions with app features and identified the most frequently used features during the first year of the intervention in a subset of 49 participants with complete app log data.

**Results:**

Of the 378 participants in the intervention group, 267 (70.6%) used the LETSGO app at least twice and were defined as app users. The median age of app users was 63 (53-70) years vs 70 (60-78) years among app nonusers (*P*<.001). App use was associated with younger age (odds ratio [OR] 0.96 per year, 95% CI 0.93‐0.99; *P*=.003), higher education (OR 2.2, 95% CI 1.1‐4.6; *P*=.03), and having ovarian cancer (OR 2.9; 95% CI 1.1‐7.5; *P*=.03). The LETSGO app included a monthly reminder for symptom self-registration, and monthly peaks in the symptom monitoring feature corresponded with the timing of this reminder. App log data from the full first year of the intervention were available for 49 participants. Among these, user engagement was highest for the physical activity and activity goal-setting features, whereas the disease information feature showed steady but less frequent use.

**Conclusions:**

Our findings indicate that LETSGO app use was associated with survivor characteristics such as age, education level, and cancer type. Considering user characteristics when tailoring mHealth apps may support user engagement in digital follow-up care and inform the development of mHealth tools that better align with survivors’ needs and preferences. Engagement with the LETSGO app during the first year of follow-up among a subset of participants with complete app log data was highest for the physical activity and activity goal-setting features. The pattern of symptom registration following reminders indicates that scheduled prompts may support regular app engagement, while steady use of the disease information feature suggests an ongoing need for accessible health information during follow-up.

## Introduction

Gynecologic cancer and its treatment can have a profound impact on survivors’ well-being, with many women experiencing physical and psychosocial late effects [1-3]. As life expectancy following cancer treatment continues to improve, the focus of care has shifted from survival alone to promoting long-term well-being and quality of life [4-7]. Consequently, follow-up care has become an important component of survivorship, not only for monitoring recurrence but also for managing late effects and providing psychological support [8-10]. At the same time, the growing population of cancer survivors places increasing pressure on the outpatient clinics, where most patients are monitored for years after treatment. This underscores the need for more sustainable follow-up strategies that meet survivors’ needs and are resource-efficient [10-12].

In this context, empowering cancer survivors to take an active role in managing their own health is increasingly recognized as a key component of future follow-up care. Such empowerment may help reduce the burden on outpatient clinics by enhancing survivors’ ability to cope with cancer-related challenges and regain control over everyday life [13]. A central aspect of empowerment is strengthening the self-management skills of cancer survivors to promote self-care, address ongoing needs after treatment, and contribute to more efficient use of health care services [13-16].

Digital interventions, particularly mobile health (mHealth) apps, have shown promise in promoting empowerment and improving quality of life among cancer survivors [17-20]. The mHealth apps are internet-based apps on mobile devices that support health-related activities. Over recent years, an increasing number of mHealth apps have been developed to meet the specific needs of patients with cancer [21-23]. These mHealth technologies can complement health care professionals by providing tailored information, enabling self-monitoring, simplifying access to health care services, supporting behavior and lifestyle changes, and thereby reducing long-term health care costs [24,25]. They can also serve as platforms for collecting patient-reported outcomes, offering valuable insights into symptoms, functional status, and overall well-being without requiring in-person visits [26,27].

The effectiveness of mHealth apps in cancer survivorship for supporting behavior and lifestyle changes, as well as symptom monitoring, depends not only on their design and content but also on regular and sustained user engagement. Research indicates that digital health interventions, including mHealth apps, are effective only when users interact with them as intended [28,29]. Nonadherence, when users fail to use the digital intervention as intended, and attrition, when they discontinue use over time, remain common challenges [30,31]. Therefore, understanding user engagement and user perspectives is essential to improve the effectiveness of mHealth apps [30,32]. Although mHealth apps are increasingly used in cancer survivorship, knowledge regarding how users interact with these technologies over time and which factors influence sustained use remains limited [29]. As mHealth apps usually include more than one feature, it is important to identify which features are most used by cancer survivors because their needs may vary depending on diagnosis and individual characteristics [33].

In the Lifestyle and Empowerment Techniques in Survivorship of Gynecologic Oncology (LETSGO) multicenter clinical trial, we evaluated a new follow-up model involving nurse-led patient education, reinforced with the LETSGO app [16]. The comprehensive LETSGO app was designed to educate patients on late effects after cancer treatment, motivate physical activity, encourage healthier lifestyle changes, facilitate symptom monitoring for recurrence detection, and provide reminders to support physical activity goals in gynecologic cancer survivorship [16].

To improve our understanding of user engagement and to support the design and development of future digital follow-up interventions that better meet survivors’ needs, this study’s primary aim was to examine associations between selected demographic and clinical characteristics and the use of the LETSGO app among gynecologic cancer survivors in follow-up care. A secondary aim was to describe 1-year use patterns, including frequency of use and the most frequently used features.

## Methods

### Study Setting and Participants

The LETSGO multicenter clinical trial recruited women (N=741) from 12 Norwegian hospitals between December 2019 and February 2023 to test a new follow-up model for routine survivorship care after treatment for gynecological cancer, aimed at enhancing health-related empowerment through education and self-management strategies, as described in the study protocol [34]. Eligible women were aged 18 years or older, had a histologically confirmed diagnosis of ovarian, endometrial, cervical, or vulvar or vaginal cancer, were able to understand Norwegian, and had no cognitive impairments. Patients were assigned to their respective group (intervention group or standard follow‐up group) based on their hospital of residence. At the intervention hospitals, women received alternating nurse-led and physician-led follow-up consultations. The nurses focused on coaching participants in empowerment techniques, reinforced by the multifunctional LETSGO app, which was introduced to the women at the initial nurse-led consultation [34]. This substudy was conducted among 378 (51%) participants in the intervention group and is based on app registration data and app log data from the first year of LETSGO app use.

### The LETSGO App

**Figure 1.**
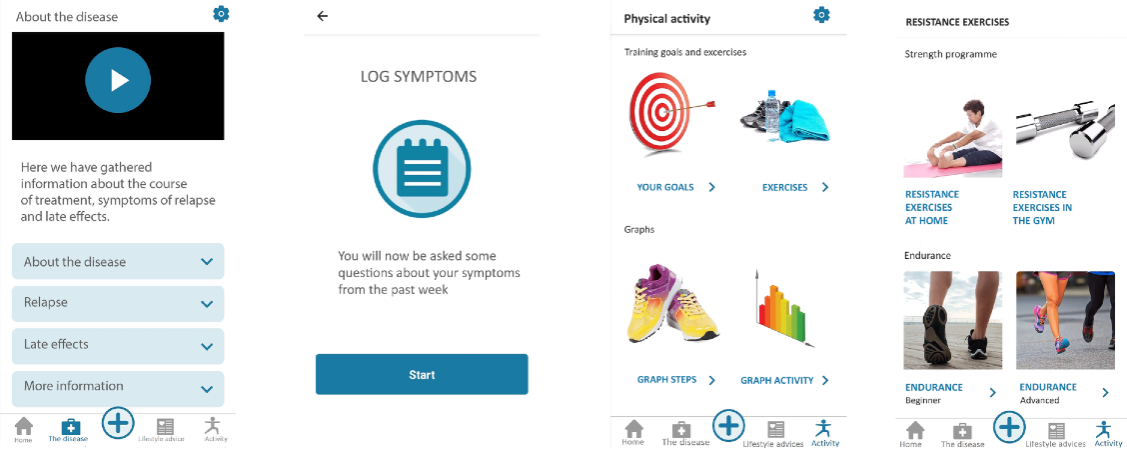
Screenshots illustrating the core features of the Lifestyle and Empowerment Techniques in Survivorship of Gynecologic Oncology (LETSGO) app (Multimedia Appendices 1-4).

The LETSGO app was developed by a multidisciplinary team of experts in the field of gynecological cancer, behavior change, exercise oncology, and IT, alongside user representatives [34]. Its primary goal was to empower women to take a more active role in their own health by providing education and motivation. Pilot testing of the LETSGO intervention and study procedures provided valuable feedback on the app’s functionality [35]. In response, technical improvements were made prior to its use in the intervention study. At the first nurse-led consultation, participants received a personal login code for the LETSGO app along with use instructions. Participants could also discuss app-related questions during follow-up consultations with nurses, including receiving help with technical or practical issues related to app use. Each participant registered their cancer type in the app to access diagnosis-specific information and saved the phone number of their outpatient clinic with direct access to the responsible nurse. The LETSGO app, accessible on smartphones and tablets, featured multiple modules, written content, links to external resources, and videos for additional guidance, as shown in Table 1 and Figure 1. Furthermore, the app offered users the option to log their own physical activities and set activity goals. Additionally, they received a wearable activity tracker to monitor their physical activity level (walking steps) [34].

**Table 1.** Content of the Lifestyle and Empowerment Techniques in Survivorship of Gynecologic Oncology (LETSGO) app.(Figure 2)

Features	Written content and videos
Contact information	Telephone number to the responsible nurse
Disease information (ovarian, endometrial, cervical, or vulvar or vaginal cancer)	Treatment Signs of recurrence Late effects Links to external information
Lifestyle information and coaching	Dietary guidance Smoking cessation Stress management Yoga and mindfulness instructions
Physical activity	Physical activity instructions Resistance exercises Endurance Activity goal setting
Electronic reminder	Electronic reminder to register physical activity Electronic reminder to register symptoms
Symptom monitoring for recurrence	10 items related to cancer recurrence

**Figure 2.**
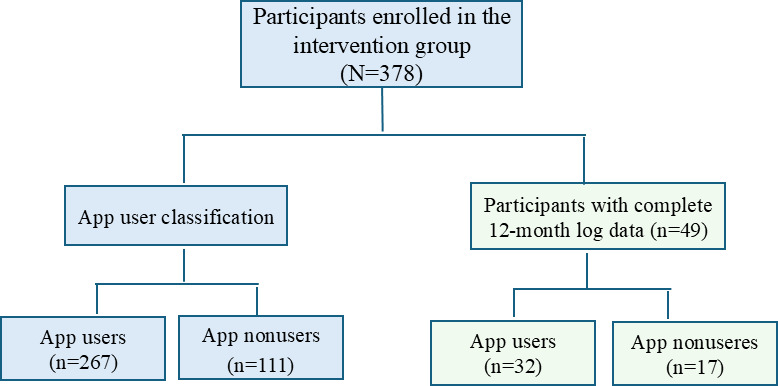
App user classification and availability of complete 12-month app log data.

The monthly monitoring of self-evaluated symptoms potentially indicating a recurrence included 10 items. These items were selected from the European Organisation for Research and Treatment of Cancer (EORTC) item library based on the most frequently reported recurrence symptoms for each type of gynecologic cancer [36]. The app sent users monthly reminders to complete the symptom monitoring questionnaire while also allowing them to complete the same questionnaire whenever needed. If their responses exceeded a predetermined threshold, an alert was triggered in the app, indicating a potential recurrence and advising them to contact the outpatient clinic. The phone number of the nurse at their outpatient clinic was automatically displayed on the screen for easy access.

### Data Collection

At baseline in the LETSGO trial, participants completed patient-reported outcome measures, including sociodemographic characteristics (ie, age, BMI, education level, and employment status). Clinical information (ie, diagnosis and treatment modality) was obtained from medical records. These data were stored in the Viedoc software (Viedoc Technologies AB), a secure electronic data capture system, and extracted for inclusion in the analysis dataset. In this study, participants’ education level was categorized as primary (≤10 years), secondary (11‐13 years), or medium or high (≥14 years), and employment status was categorized as 0=no or 1=yes. Cancer diagnosis was categorized as endometrial, ovarian, cervical, or vulvar cancer. Treatment modality was categorized into three groups: (1) surgery only, (2) surgery plus chemotherapy, and (3) chemotherapy plus radiotherapy (with or without surgery) and other treatment combinations. Because of the low number of participants receiving chemotherapy and radiotherapy and other treatment combinations, these were combined into a single category.

All app data included in this study (app registration data and app log data) from the first year of the intervention were retrieved from the Services for Sensitive Data (TSD) platform at the University of Oslo, a secure system for handling sensitive research data. The app data included participant registrations in the physical activity, activity goal setting, and symptom monitoring features.

In addition, app log data capturing use frequency, measured as the number of clicks, were available for the physical activity, activity goal setting, disease information, and lifestyle information and coaching features. However, due to a miscommunication between the research team and the app developers regarding data storage, app log data were not stored from the beginning of the intervention period. Once this was identified, app log data collection was implemented. Consequently, app log data covering the full 12-month intervention period were available for only 49 participants. The analysis of app-use frequency over 12 months was limited to this subset of 49 participants.

App users were defined as those participants who, during the first year of the intervention, registered symptoms (symptom monitoring) at least twice and reported weekly physical activity (physical activity) and/or activity goals (activity goal setting) at least twice. There is currently no consensus on how engagement thresholds in digital health interventions should be operationalized, and our definition was pragmatically chosen to distinguish participants with repeated use of both features from those who did not meet this threshold, while also avoiding unnecessary exclusion of participants with low-frequency engagement [28,30].

### Statistical Analyses

All statistical analyses were performed using SPSS Statistics (version 30; IBM Corp).

Participant characteristics were described using descriptive statistics. Continuous variables were presented as medians and ranges, while categorical variables were presented as counts and percentages. Group differences were assessed using the Mann-Whitney *U* test for continuous variables and the Pearson chi-square test for pairs of categorical variables.

Multiple logistic regression analyses were conducted to identify participant characteristics associated with app use and to examine which groups were more likely to use the app. App use (coded as 0=no and 1=yes) was the dependent variable. Variables considered clinically relevant and potentially associated with app use, including demographic (eg, age, education level, and employment status) as well as clinical characteristics (ie, diagnosis and treatment modality), were entered as possible predictive factors. These independent variables were entered into the analysis simultaneously and treated as covariates, allowing the association between each variable and app use to be estimated while controlling for the others. Multicollinearity between independent variables was assessed, and no evidence of problematic multicollinearity was identified. Odds ratios (ORs) with 95% CIs were calculated to assess the strength of association.

Weekly symptom registrations over the first year of the intervention for all participants (N=378) were summarized to describe overall engagement with the symptom monitoring feature.

A subanalysis of the 49 participants with complete app log data covering the full 12-month intervention period was conducted to assess the frequency of user interactions with the key app features (physical activity, activity goal setting, disease information, and lifestyle information and coaching), and to identify which app features were most frequently used.

The app log data captured the number of user clicks within each feature. Results are presented as bar and line graphs. Differences in demographic and clinical characteristics between participants with complete app log data covering the full 12-month intervention period and those without were assessed using the Mann-Whitney *U* test for continuous variables and the Pearson chi-square test for categorical variables. All tests were 2-sided, and *P*<.05 was considered statistically significant. All results are considered exploratory; therefore, no correction for multiple testing was applied.

### Ethical Considerations

The LETSGO study (ClinicalTrials identifier NCT04122235) was approved by the Regional Committee for Medical and Health Research Ethics (REK 2019/11093) and the data protection officers at participating hospitals. Written informed consent covering all aspects of the LETSGO study was obtained from the participants prior to inclusion. The data from the LETSGO app were stored on the TSD platform at the University of Oslo, and sociodemographic and clinical data were stored in Viedoc software.

## Results

### Characteristics of the Participants

Of the 378 participants included in the analysis, 267 (70.6%) were defined as app users. An overview of participant classification and availability of complete 12-month app log data is presented in Figure 2. Some baseline variables were missing for 39 participants. There were no statistically significant differences between those with and without missing data regarding age (*P*=.23), treatment modality (*P*=.38), and type of diagnosis (*P*=.26). Of those with missing data, 33.3% (13/39) were app users. The median age of all participants was 65 years, and app users were significantly younger than app nonusers (median age 63 vs 70 years). App users had significantly higher education and were more often employed compared with app nonusers (Table 2). Endometrial cancer was the most common diagnosis (221/378, 58.5%).

**Table 2.** Baseline characteristics of the participants (N=378).

	Total group (n=378)	App users^a^ (n=267)	App nonusers^b^ (n=111)	*P* value
Age (years), median (IQR)	65 (54-72)	63 (53-70)	70 (60-78)	<.001
BMI (kg/m^2^), median (IQR)	27 (24-32)	27 (24-32)	28 (24-32)	.76
Missing, n (%)	41 (10.8)	14 (5.2)	27 (24.3)	—^c^
Diagnosis, n (%)				.18
Endometrial cancer	221 (58.5)	151 (56.6)	70 (63.1)	
Ovarian cancer	92 (24.3)	73 (27.3)	19 (17.1)	
Cervical cancer	58 (15.3)	39 (14.6)	19 (17.1)	
Vulvar cancer	7 (1.9)	4 (1.5)	3 (2.7)	
Treatment modality, n (%)				.29
Surgery only	213 (56.3)	145 (54.3)	68 (61.3)	
Surgery plus chemotherapy	121 (32)	92 (34.5)	29 (26.1)	
Chemotherapy plus radiotherapy and other combinations^d^	44 (11.6)	30 (11.2)	14 (12.6)	
Education, n (%)				<.001
Primary	50 (13.2)	28 (10.5)	22 (19.8)	
Secondary	156 (41.3)	124 (46.4)	32 (28.8)	
Medium or high	133 (35.2)	102 (38.2)	31 (27.9)	
Missing	39 (10.3)	13 (4.9)	26 (23.4)	
Employment, n (%)				<.001
Yes	120 (31.7)	103 (38.6)	17 (15.3)	
No	219 (57.9)	151 (56.6)	68 (61.3)	
Missing	39 (10.3)	13 (4.9)	26 (23.4)	

a App users: ≥2 registrations of symptoms and ≥2 registrations of weekly physical activity or activity goals during the first year of follow-up.

b App nonusers: Participants who did not meet the definition of app users.

c Not applicable.

d Participants receiving chemotherapy plus radiotherapy and other treatment combinations were grouped due to low numbers.

### Factors Associated With App Use

Participants with missing data on variables included in the logistic regression model were excluded from the analysis, resulting in 337 complete cases included in the adjusted model.

Women with vulvar cancer were excluded from the regression analysis due to the low number of participants in this diagnosis group.

In the multiple logistic regression analysis, adjusted for age, education level, employment status, type of diagnosis, and treatment modality, increasing age was associated with lower odds of app use. For each additional year of age, the odds for app use were approximately 4% lower (OR 0.96 per year, 95% CI 0.93‐0.99; *P=.*003). Younger participants had higher odds of app use, with a 10-year decrease in age being associated with approximately 50% higher odds of using the app. Participants with secondary education had more than 2-fold higher odds of app use compared with those with primary education (OR 2.23, 95% CI 1.09‐4.56; *P=*.03). Participants diagnosed with ovarian cancer had higher odds of app use compared with those diagnosed with cervical cancer (OR 2.93, 95% CI 1.14‐7.53; *P=*.03). Similarly, participants diagnosed with endometrial cancer had higher odds of app use compared with those diagnosed with cervical cancer (OR 3.31, 95% CI 1.16‐9.49; *P=.*03). Other variables, including treatment modality and employment status, were not associated with app use (Table 3).

**Table 3.** Factors associated with app use based on multiple logistic regression analysis.

Variables	OR (95% CI)		*P* value
Age	0.96 (0.93‐0.99)		.003
Education			
Primary	Reference		—^a^
Secondary	2.23 (1.09‐4.56)		.03
Medium or high	1.48 (0.69‐3.18)		.31
Employment status			
Yes	1.70 (0.81‐3.56)		.16
Cancer type			
Cervical	Reference		—
Ovarian	2.93 (1.14‐7.53)		.03
Endometrial	3.31 (1.16‐9.49)		.03
Treatment modality			
Surgery only	Reference		—
Surgery plus chemotherapy	1.56 (0.79‐3.12)		.20
Chemotherapy plus radiotherapy and other combinations	1.43 (0.53‐3.83)		.48

a Not applicable.

### Registration in the Symptom Monitoring Feature

**Figure 3.**
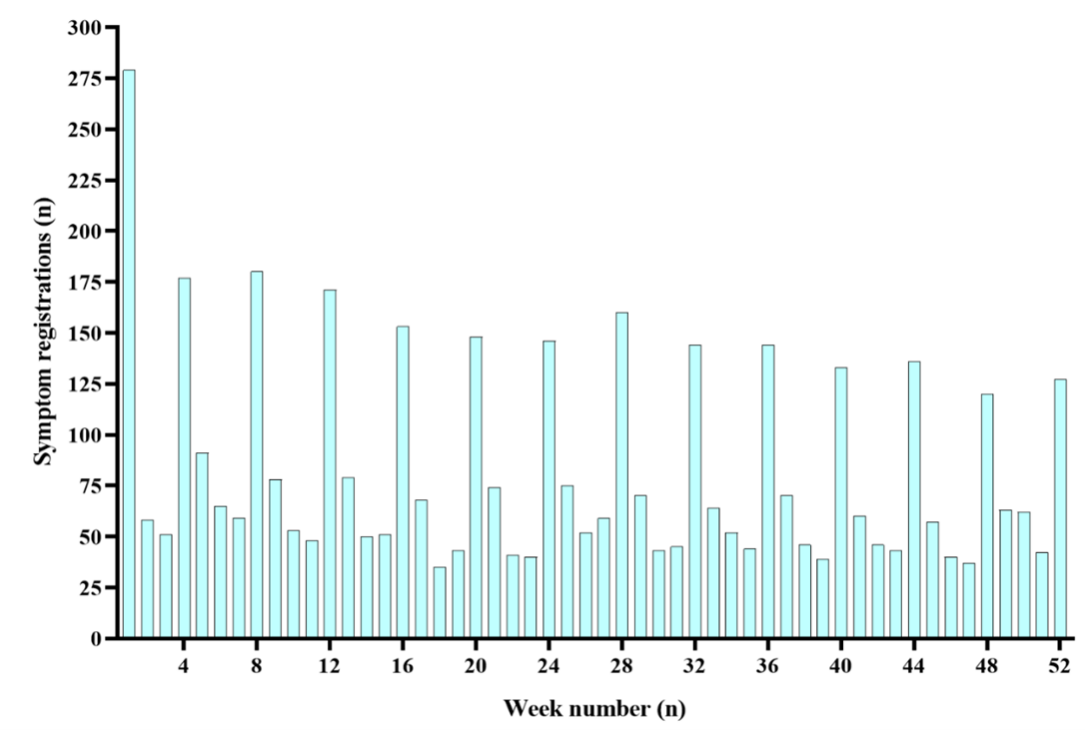
Weekly number of symptom registrations over the 52-week follow-up period.

Of the 378 participants, 310 (82%) registered symptoms using the symptom monitoring feature at least once, including participants who did not meet the predefined criteria for app use. Symptom registration was most frequent in the first weeks after inclusion, followed by a decline and stabilization over the 1-year period (Figure 3). We observed monthly peaks in registrations that corresponded to the app reminder being sent once per month. However, participants also registered symptoms between reminders, indicating engagement with the feature outside the scheduled prompts.

### App Feature Use Over 12 Months for 49 Participants With Complete Log Data

**Figure 4.**
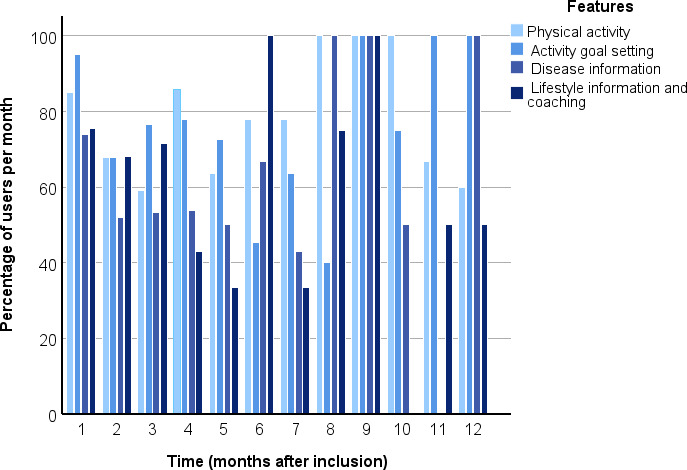
Proportion of users engaging with each app feature at least once per month during the 12-month follow-up period (n=49).

**Figure 5.**
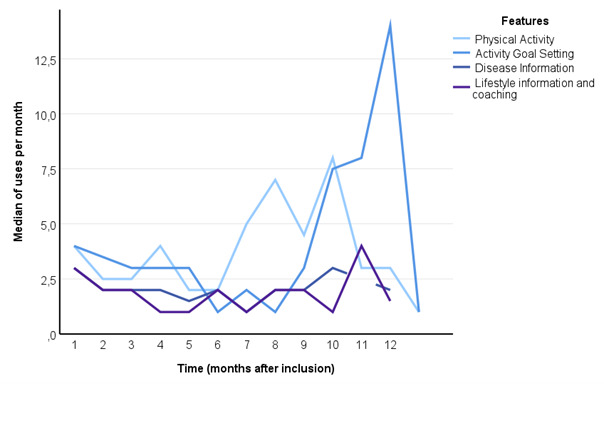
Median monthly use frequency of each app feature during the 12-month follow-up period (n=49).

A subanalysis of frequencies of app use among 49 participants over the first year of intervention showed that the proportion of users who interacted with each feature at least once per month was the highest for physical activity (n=39, 79.6%), followed by activity goal setting (n=29, 59.2%), while disease information and lifestyle information and coaching showed lower but relatively consistent engagement (n=10-20, 20.4%‐40.8%) over time (Figure 4). The median number of uses per month, defined as the number of individual participant registrations within app features, also indicated that physical activity and activity goal setting features were used most frequently (Figure 5). Among the 49 participants with complete log data, the median of app-use frequency was 104 (IQR 54‐301; range 16‐10,767). There were no statistically significant differences between participants with app log data covering the full intervention period (n=49) and those without (n=329) regarding age, BMI, education level, employment status, diagnosis, and treatment modality (data not shown).

Together, Figures 2 to 4 illustrate different aspects of app engagement during the first year of the intervention, including symptom registrations over time, the proportion of users engaging with different app features, and monthly use frequency.

## Discussion

### Principal Findings

In this observational study of 378 gynecologic cancer survivors participating in the LETSGO trial who had access to the LETSGO app as part of the intervention, app use was more common among younger women, those with secondary education, and women diagnosed with ovarian cancer. Symptom registration was highest shortly after inclusion and stabilized over time with monthly peaks corresponding to the app’s reminder schedule. In a subanalysis of 49 app users with complete log data, engagement was highest for physical activity and activity goal setting features, while use of the disease information feature remained steady but less frequent.

The inverse association between age and app use aligns with previous studies showing that age may influence engagement with digital technologies, including mHealth technologies [37-40]. An umbrella review of 46 systematic and scoping reviews found that most digital interventions were effective regardless of age, whereas results on actual use and engagement showed greater variation [41]. Our findings align with this, indicating that although digital interventions may be effective across age groups, their uptake and engagement still differ by age. This may reflect differences in digital skills, familiarity with technology, or digital health literacy [42]. Older individuals may experience barriers related to digital health interventions, including lower digital competence, reduced confidence in using technology, lower digital health literacy, and less encouragement from health care providers [43,44].

Previous research [41] has highlighted that education has often been overlooked in existing research, even though it may contribute to the digital gap. Educational level may reflect both digital skills and general health awareness, which are important factors for engaging with digital health interventions. Earlier studies have reported higher app use among individuals with higher (tertiary) education [45,46]. In our study, however, app use was higher among women with secondary education compared with those with primary education. Among women with higher education, there was also a tendency toward higher app use compared with those with primary education, although this difference was not statistically significant. A possible explanation for the lack of a stronger association could be that women with higher education already have greater access to digital health technologies in general, which could reduce their need for this specific app [47]. However, this hypothesis was not directly examined in our study. This disparity underlines the importance of examining how education levels influence both use and sustained engagement with digital interventions in future research.

We found that women diagnosed with ovarian and endometrial cancer had higher odds of app use compared with women with cervical cancer. This finding may reflect differences in the disease trajectory, treatment intensity, and follow-up needs across diagnostic groups. One possible explanation for the observed association among women with ovarian cancer, suggested by previous research, is that ovarian cancer is often associated with greater symptom burden, longer treatment trajectories, and a perceived need for closer follow-up [48,49], which could make digital tools for symptom tracking and support more relevant and useful in their care routines [50].

Regular symptom monitoring may indicate that participants found the feature useful, which is supported by findings from a study reporting higher engagement when patients perceived symptom monitoring as relevant and meaningful for managing their symptoms [51]. In our study, the monthly peaks following reminders suggest that scheduled prompts support sustained engagement among participants. Some symptom registrations occurred outside the immediate monthly reminder pattern. However, the available data do not allow us to determine whether these registrations were self-initiated or delayed responses to reminders.

This finding aligns with previous research showing that structured symptom monitoring can encourage sustained app use over time [52]. Nevertheless, the distinction between reminder-driven and self-initiated use may be important to consider when designing future digital follow-up apps. Balancing structured guidance, such as reminders, with the opportunity for users to engage when they find it relevant could help maintain user engagement over time while ensuring that users feel supported without becoming dependent on reminders.

Although the impact of patient-directed alerts in the LETSGO app was not examined in our study, such alerts may still play a role in shaping how patients engage with and respond to the symptom monitoring feature. In the LETSGO app, patient-directed alerts are generated when symptom reports exceed predefined threshold values and prompt patients to take action, such as contacting a health care provider. Unlike many mHealth apps that notify clinicians, alerts in the LETSGO app are one-way and directed only to patients. Previous studies have shown that patient-directed symptom alerts can support earlier action and enhance patient self-management [26]. Sending alerts to patients rather than clinicians may also reduce the burden on health care providers [26]. This design supports patient empowerment approaches that encourage patients to take a more active role in their own health, which is one of the goals of mHealth interventions [32].

The high engagement with physical activity–related features in the subanalysis of 49 app users may indicate that physical activity and goal setting are particularly important for some participants in this subgroup. However, because this analysis was based on a small sample, these use patterns should be interpreted with caution and not generalized to the entire intervention group. Within these limitations, our findings are consistent with previous work highlighting the central role of physical activity features in digital interventions for cancer survivors [53,54], and they may reflect that such features foster feelings of progress, help users regain a sense of control, and support self-management during recovery [55]. This is further supported by a study [56] that found that physical activity tracking and goal-oriented features were important drivers of motivation and engagement in physical activity in a mHealth intervention for breast cancer survivors.

The steady use of the disease information feature indicates an ongoing need for accessible and reliable information that supports participants’ understanding of their condition. This observation should be interpreted in light of previous research showing that digital health literacy is often limited and influenced by factors such as age, gender, and education level [57]. This underscores the need to develop cancer-related information that is easily accessible and user-friendly across digital health platforms, including mHealth apps.

### Study Strengths and Limitations

Strengths of this study include a large sample size (N=378), which allowed for robust analysis and increased the precision of the estimated associations between app use and participant characteristics. Because Norway ensures equal access to health care and consistent cancer treatment nationwide, our unselected, multihospital sample strengthens the external validity of the findings. Finally, app use was measured based on app log data rather than self-reports, thereby reducing possible recall bias.

However, the study has several limitations. The criteria for app use were defined by the minimum user threshold and therefore reflected minimal active use rather than sustained long-term engagement. Definitions of engagement in digital health research vary and are measured in different ways, with no clear standard for how engagement thresholds should be operationalized [28,30]. The results of this study reflect only 1 year of access to the LETSGO app, and engagement patterns over a longer period may differ. Although analyses were adjusted for demographic and clinical variables, unmeasured factors such as digital literacy or attitudes toward technology may have influenced app use. Missing data in some baseline variables may have affected the findings and introduced potential bias, as the logistic regression analyses were based on complete cases only. In addition, women with vulvar cancer were too few to be included in the regression analysis, precluding the possibility of drawing any conclusions for this diagnosis group. Analyses of app-use frequency over 12 months were limited to the 49 participants with complete log data. Although participants with and without complete log data did not differ in measured baseline characteristics, unmeasured technical or implementation-related factors may still have influenced log data availability. The small number of participants with complete log data also limited the precision of the estimates. Therefore, findings related to app feature use and use frequency should be interpreted with caution.

### Implications for Practice

The observed variation in app use across demographic and clinical groups indicates that a one-size-fits-all digital intervention may not be sufficient. Addressing these factors when developing interventions can help promote equitable and sustained use across diverse user groups and enhance engagement among groups less likely to use the app. In clinical practice, this highlights the need for targeted onboarding and user support, such as motivational guidance and technical assistance, tailored to the needs of different patient groups to ensure equal access to and benefit from app-based support in follow-up care.

Although app log data for the full first year were available only for a subset of the participants, the highest engagement with the physical activity and activity goal setting features among these participants suggests that encouraging regular physical activity and goal setting may be perceived as supportive by survivors, helping them monitor their activity levels and maintain a sense of control during recovery. Symptom alerts delivered directly to survivors may support earlier self-initiated contact with health care providers, thereby potentially facilitating timely clinical assessment when needed.

### Conclusions

App-use patterns indicate differences in engagement across app features and participant characteristics. Findings from this study underscore the need to consider demographic and clinical characteristics when implementing digital tools in follow-up settings to ensure equal participation and avoid disparities in follow-up care. Overall, this study provides insight into the potential relevance of the LETSGO app in routine follow-up after cancer treatment, suggesting that such mHealth approaches may be applicable across different gynecologic cancer diagnoses. These findings may also inform the development and implementation of future mHealth tools designed to support cancer survivors in routine follow-up care.

## Supplementary material

10.2196/89918 Multimedia Appendix 1 Information about the disease, treatment, and late effects.

10.2196/89918 Multimedia Appendix 2 Symptom registration feature.

10.2196/89918 Multimedia Appendix 3 Physical activity feature.

10.2196/89918 Multimedia Appendix 4 Exercise program feature.

## Abbreviations

EORTC: European Organisation for Research and Treatment of Cancer
LETSGO: Lifestyle and Empowerment Techniques in Survivorship of Gynecologic Oncology
mHealth: mobile health
OR: odds ratio
TSD: Services for Sensitive Data
