# Supplementary figures and images for: Factors Associated With the Use of the Lifestyle and Empowerment Techniques in Survivorship of Gynecologic Oncology (LETSGO) mHealth App in Routine Follow-Up After Gynecologic Cancer Treatment: Observational Study
Source: JMIR Cancer. 2026 Jul 31;12:e89918. doi: 10.2196/89918 (PMC13426126; doi:10.2196/89918)

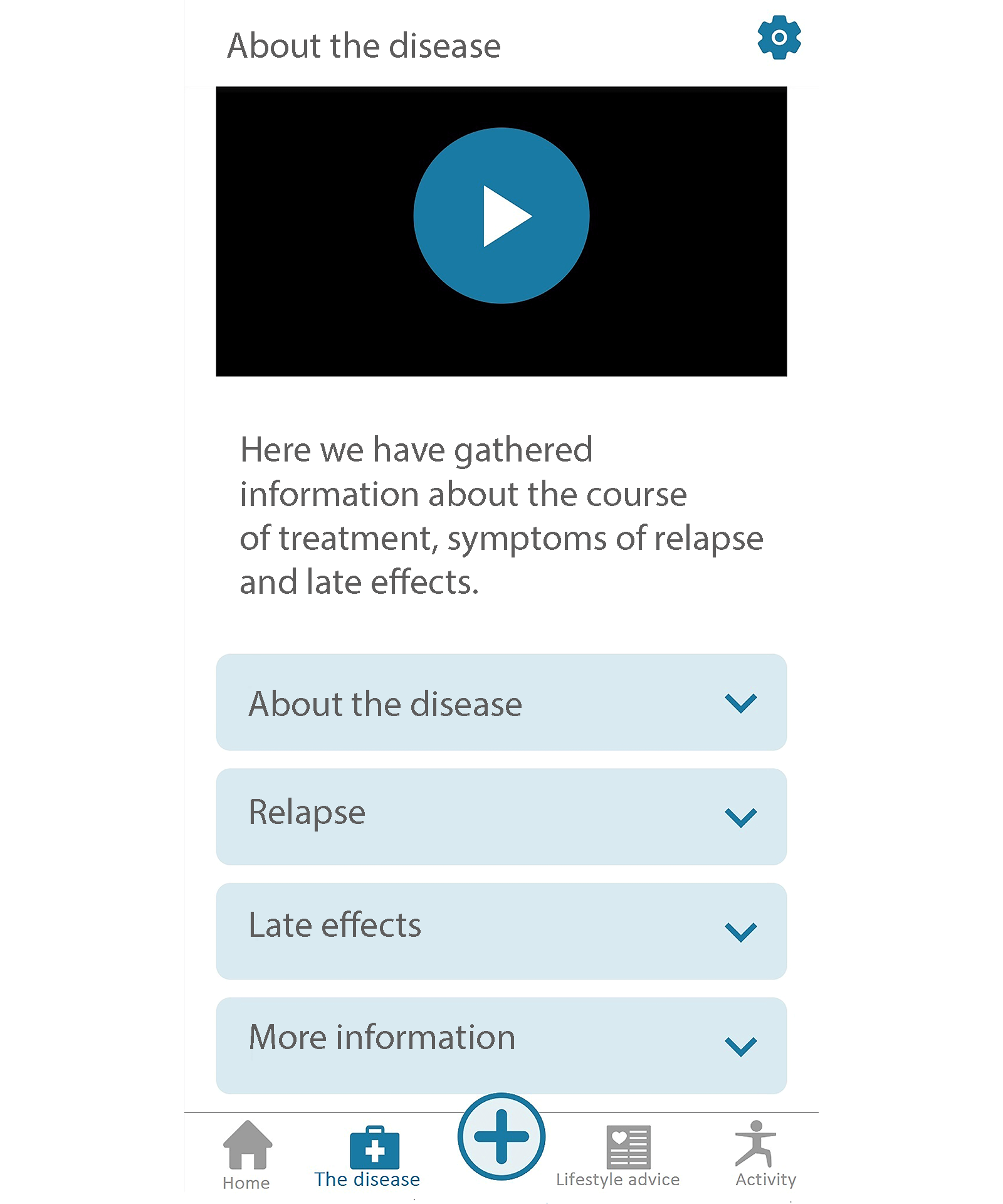

Supplement: Multimedia Appendix 1 [file cancer-v12-e89918-s001.png]

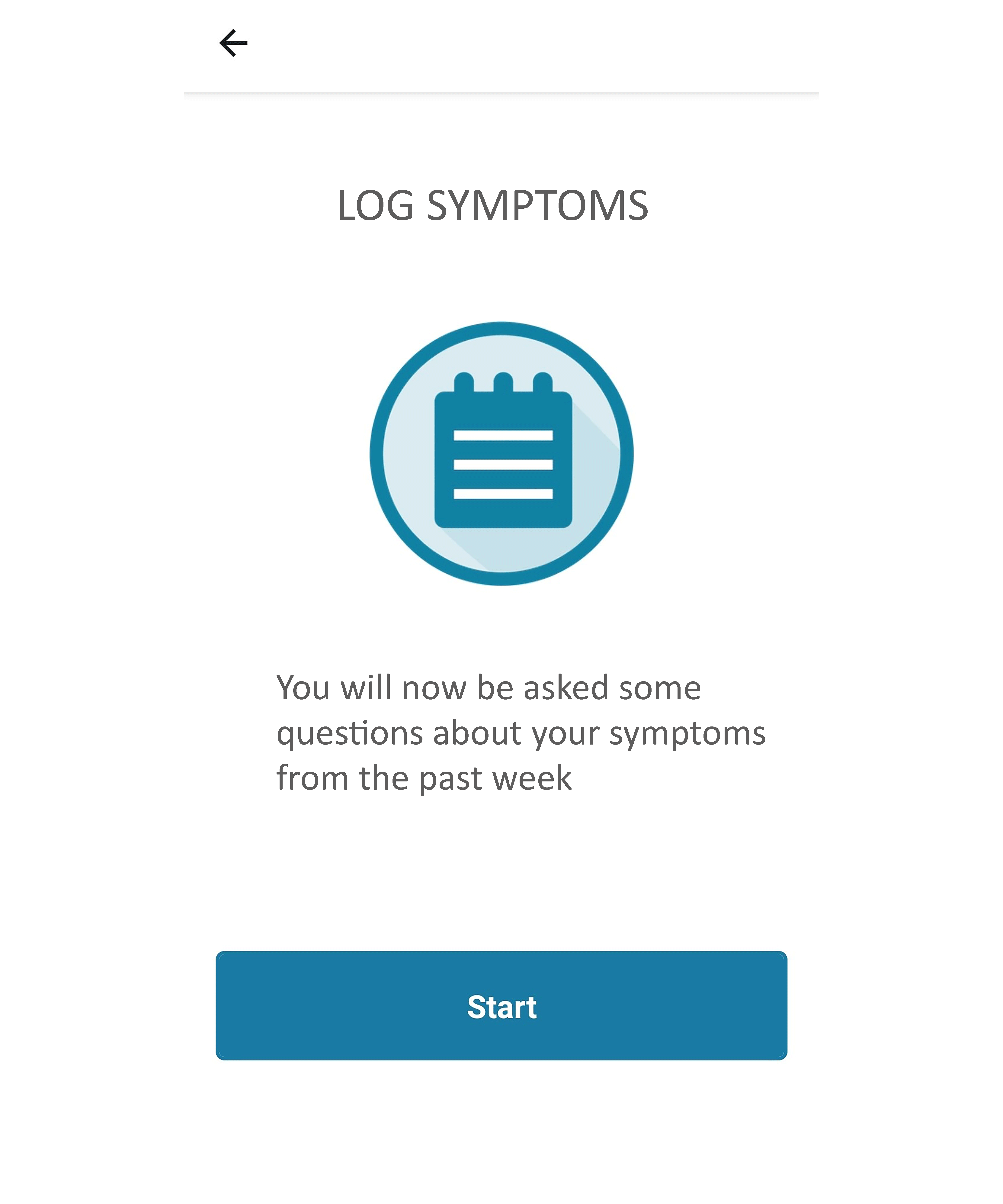

Supplement: Multimedia Appendix 2 [file cancer-v12-e89918-s002.png]

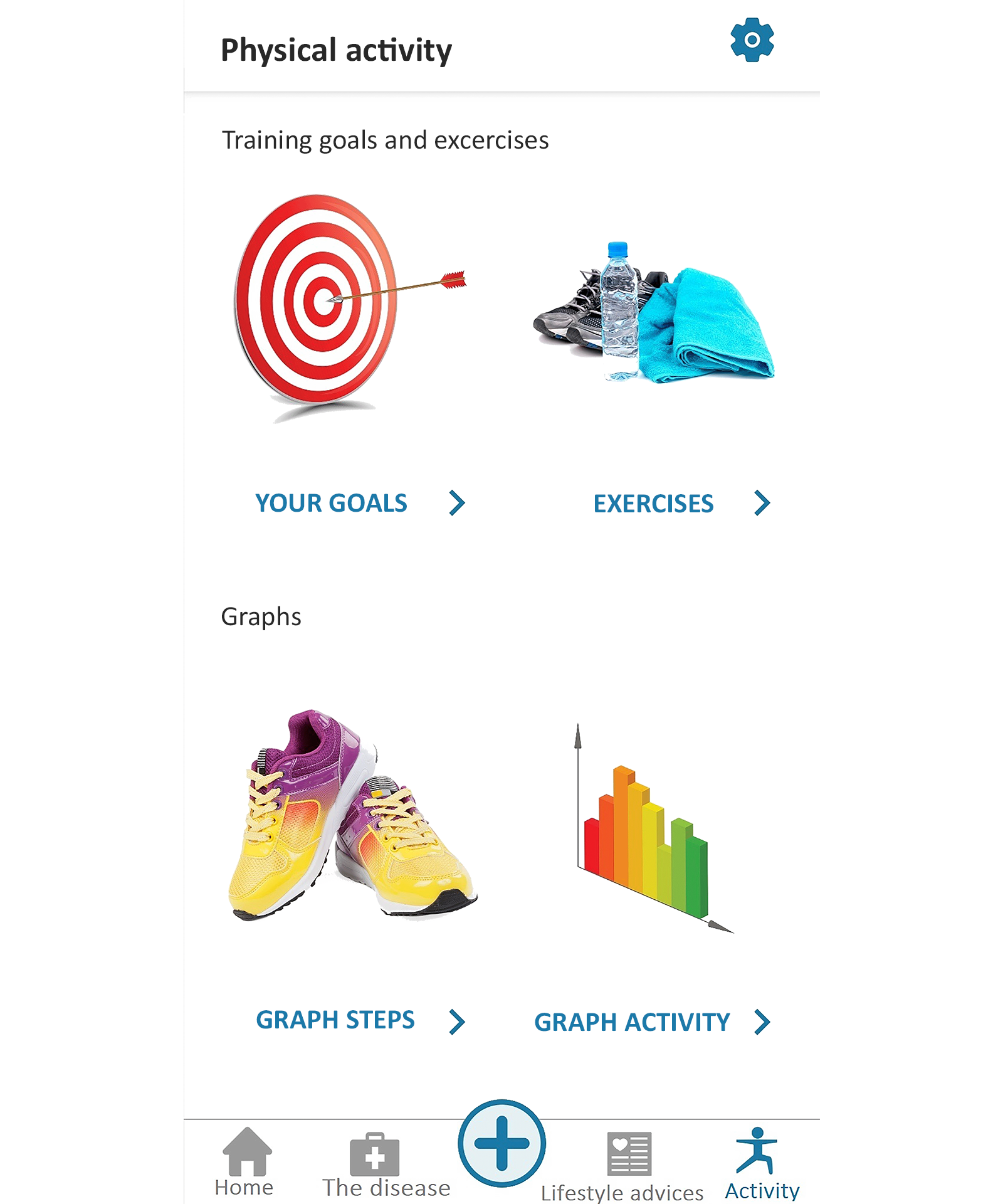

Supplement: Multimedia Appendix 3 [file cancer-v12-e89918-s003.png]

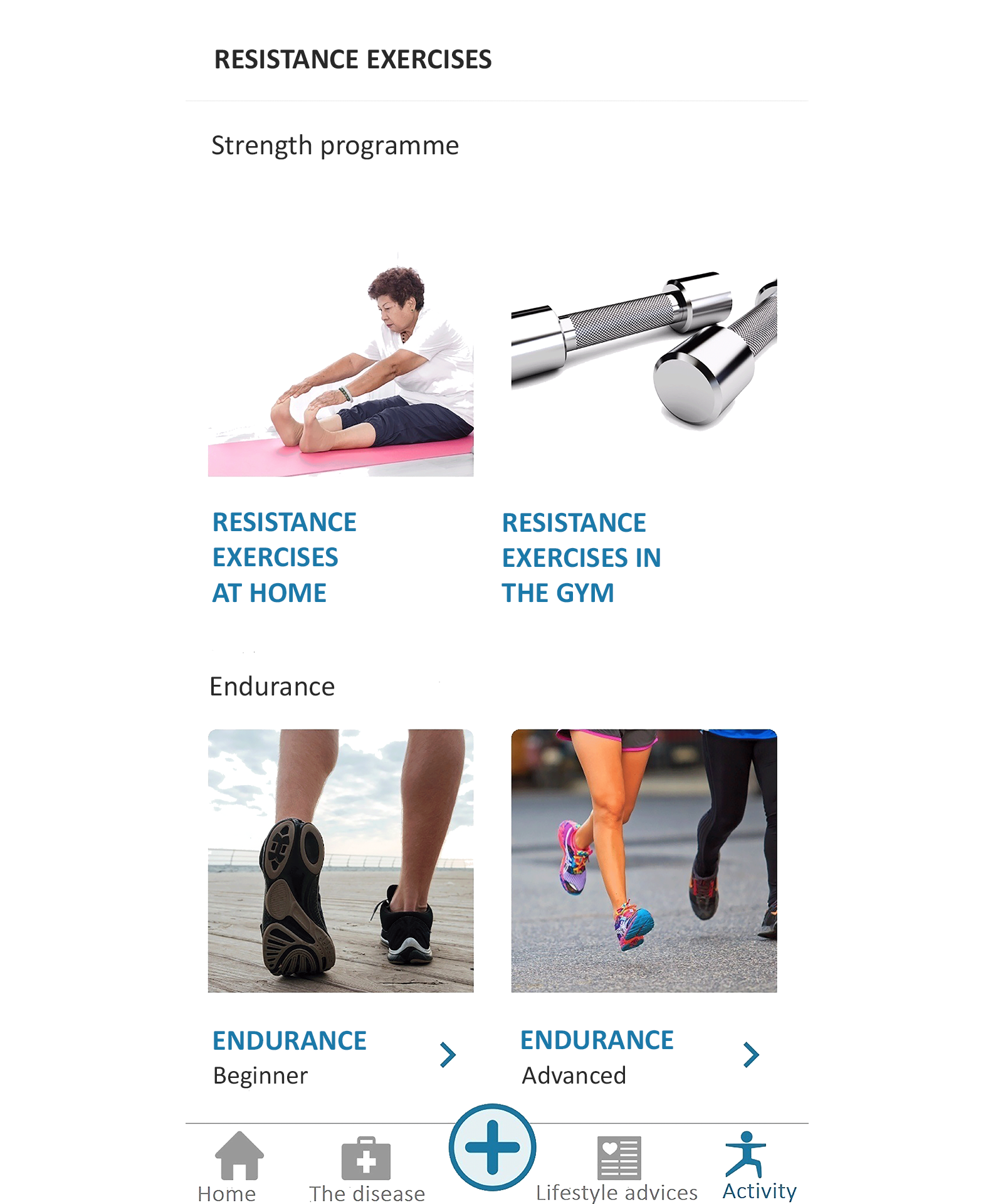

Supplement: Multimedia Appendix 4 [file cancer-v12-e89918-s004.png]
